# Hereditary Angioedema With Normal C1 Esterase Inhibitor: A Case of a Late Diagnosis in a 48-Year-Old Male

**DOI:** 10.7759/cureus.93759

**Published:** 2025-10-03

**Authors:** Natalie Elazar, Marc M Kesselman

**Affiliations:** 1 Osteopathic Medicine, Nova Southeastern University Dr. Kiran C. Patel College of Osteopathic Medicine, Davie, USA; 2 Rheumatology, Nova Southeastern University Dr. Kiran C. Patel College of Osteopathic Medicine, Davie, USA

**Keywords:** bradykinin-mediated angioedema, c1 esterase inhibitor, diagnostic delay, hae with normal c1 inhibitor, hereditary angioedema (hae), late diagnosis, recurrent swelling

## Abstract

Hereditary angioedema (HAE) is a rare disorder characterized by recurrent, non-pitting edema affecting the mucosal, submucosal, or subcutaneous tissues. A rarer subtype, HAE with normal C1 esterase inhibitor (HAE-nC1-INH), lacks universally accepted biomarkers.

We present a 48-year-old male with a history of Epstein-Barr virus (EBV) and hyperlipidemia, who experienced recurrent episodes of angioedema involving the lips, face, throat, hands, feet, and genitalia. Prior treatments, including corticosteroid injections and oral prednisone, were ineffective. The patient denied any relevant family history and reported symptom exacerbations, potentially triggered by stress. Laboratory evaluation included antinuclear antibody (ANA), C1-INH functional and protein levels, C1Q, total complement, rheumatoid factors, Smith (SM) antibodies, and anti-double-stranded DNA (anti-dsDNA) antibodies. The patient was prescribed icatibant for acute flares and berotralstat for prophylaxis.

This case highlights the diagnostic challenges of HAE-nC1-INH, a subtype not responsive to standard treatment with antihistamines or corticosteroids. Although this patient did not experience life-threatening airway compromise, delayed diagnosis of this condition can lead to severe outcomes. Increased awareness of HAE-nC1-INH among clinicians is essential to improve timely diagnosis and appropriate treatment. This case underscores the importance of differentiating HAE-nC1-INH from other forms of angioedema to prevent misdiagnosis.

## Introduction

Angioedema is characterized by asymmetrical, non-pitting swelling - also referred to as edema - that affects the mucosal, submucosal, or subcutaneous tissues [1]. There are two main causes of angioedema: bradykinin-mediated and histamine-mediated [1]. Histamine-mediated angioedema is often triggered by allergens or medications. Common triggers include certain foods or drugs, like ACE inhibitors (ACEi), and swelling may occur in areas such as the face, extremities, genitalia, or upper airway. ACEi prevent the breakdown of bradykinin by blocking angiotensin-converting enzymes, leading to increased bradykinin levels [2]. Airway swelling can be life-threatening due to the risk of obstruction, subsequent respiratory failure, or even death [3]. A rare subtype of hereditary angioedema (HAE), known as HAE with normal C1 esterase inhibitor (HAE-nC1-INH), is diagnosed based on exclusion and has no specific blood markers available for diagnosis. A normal C1-INH level - as well as a history of recurrent angioedema without resolution, the presence of mast cell inhibitors, and no urticaria - suggests this rare disease [4]. 

HAE is considered a rare condition, affecting approximately 1 in 50,000 people worldwide [3]. The average age of diagnosis is around 20 years old, with the first symptoms typically starting in adolescence [5]. HAE due to C1-INH deficiency is a genetic condition caused by mutations in the SERPING1 gene. These mutations result in reduced production or function of C1-INH, a glycoprotein produced in the liver. This protein plays a key role in regulating the complement system, which, in healthy individuals, helps the body respond to pathogens we encounter [6].

The complement cascade is a highly regulated immune response that, when dysregulated, contributes to inflammation and vascular permeability. In HAE, the lack of C1-INH leads to increased activation of the kallikrein-kinin system through activation of Factor XII. This results in the excess release of bradykinin, a chemical that causes vasodilation and increases vascular permeability by triggering the release of substances like nitric oxide and prostacyclin. The end result is angioedema [7]. Concurrently, if the complement system is too weak or under-regulated, the body may become more vulnerable to infections. It’s the careful balance of activators and regulators that keeps the system functioning properly.

Recognizing triggers early is critical for patient safety, as is identifying the correct underlying mechanism. This is why it’s important to differentiate between C1-INH deficiency and histamine-induced angioedema. This case report explores a unique presentation of a late diagnosis of HAE-nC1-INH and highlights the challenges associated with its identification and management.

## Case presentation

**Figure 1:**
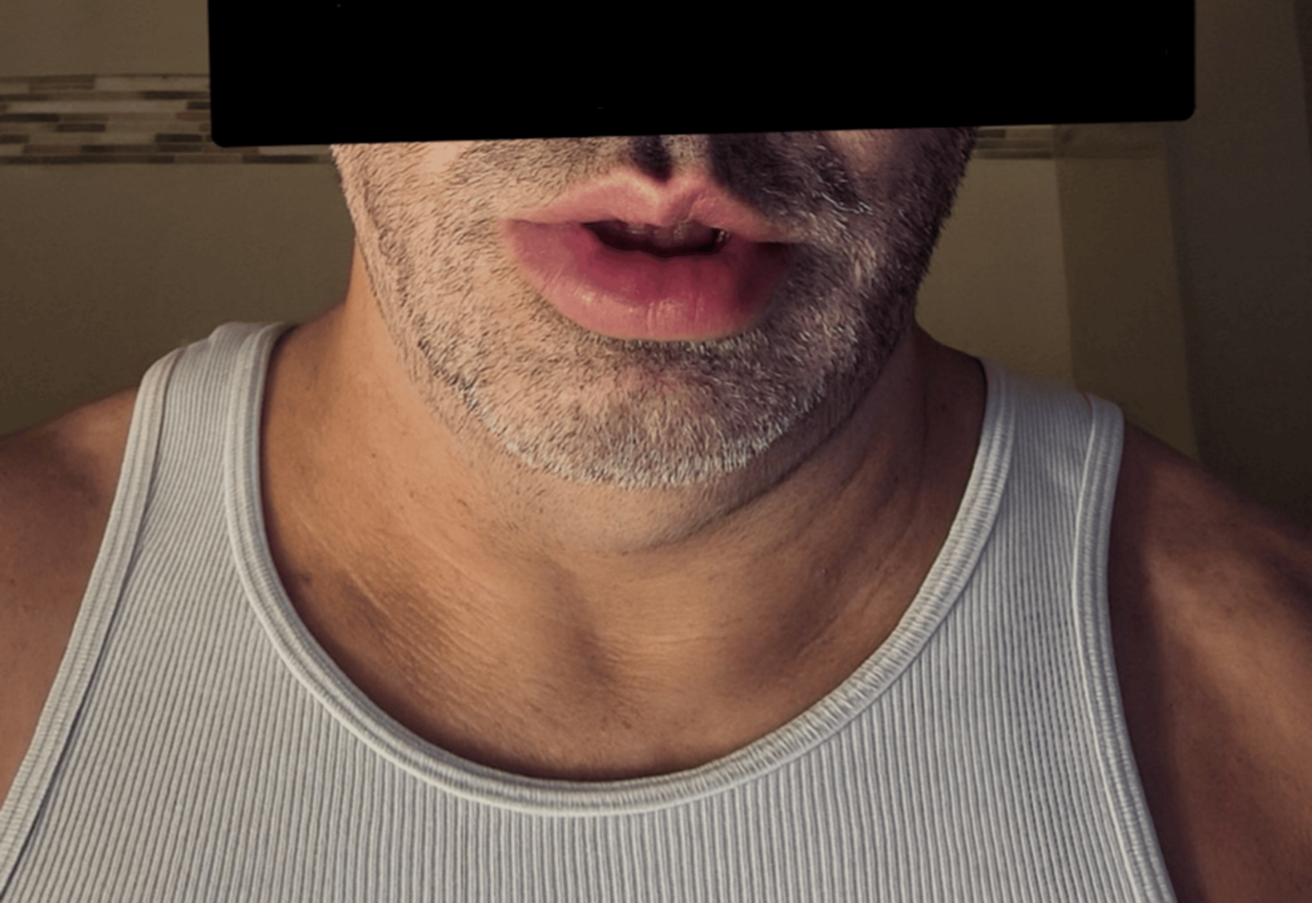
Patient A with lower lip swelling during an acute angioedema flare.

**Figure 2:**
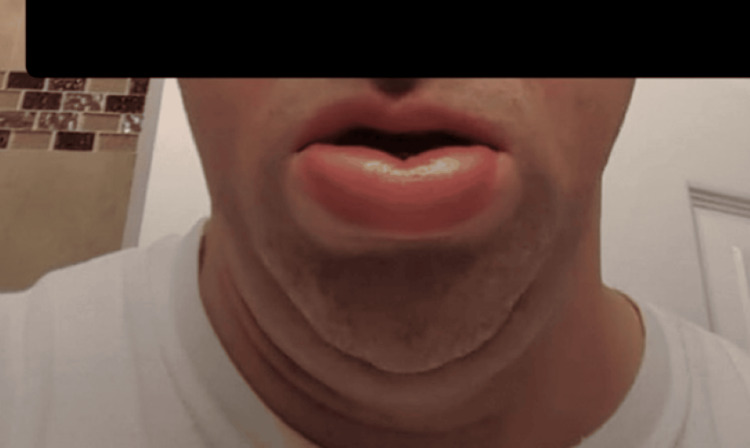
Patient A with lower lip swelling during an acute angioedema flare.

Patient A is a 48-year-old male with a history of Epstein-Barr virus (EBV), diagnosed in 2008, and hyperlipidemia, who presents with recurrent angioedema of his lower lip, face, throat, and extremities - including hands, feet, and genitals - as shown in Figures 1-2. Patient A reported that the swelling had been intermittent and started 10 years ago. During his first flare, a comprehensive diagnostic workup was performed, including assessment of C1 esterase inhibitor levels, all of which yielded results within normal limits. Patient A was initiated on antihistamine therapy for symptom management; however, he continued to experience near-daily flare-ups. He reported frequent visits to the emergency department for acute symptom exacerbations of lip enlargement and denied any identifiable or consistent triggers.

Notable symptomatic episodes include significant digital swelling, resulting in constriction around the patient's wedding ring - raising concerns for potential vascular compromise and possible ring removal. Another instance involved isolated genital edema. The urologist hypothesized that the swelling may have been due to a spider bite and administered an intramuscular corticosteroid injection, then initiated an oral prednisone regimen. Due to a lack of symptom improvement with the prescribed medications, he elected to go to the emergency department, where another corticosteroid injection was administered, and oral prednisone was continued, despite the initial treatment being ineffective. Patient A reported that symptom exacerbations may have been stress-related and denies any known family history relevant to the condition at this time.

While HAE is known to be triggered by viral infections, the patient’s EBV infection was contracted years before the onset of symptoms. Unfortunately, no further workup was performed at the time of EBV diagnosis, nor was any treatment initiated. Although there can be speculation regarding a potential association between these conditions, no evidence supports such a link in this case.

A review of the patient’s pertinent medications taken prior to the visit includes fenofibrate for dyslipidemia, gabapentin for chronic wrist pain, and ibuprofen, taken in an attempt to manage his swelling. Allergies noted on the chart include levofloxacin and penicillin. Reactions to these medications include hives and difficulty breathing. All reviews of systems were negative in the office. Vitals showed a blood pressure of 120/77 mmHg, pulse rate of 90 bpm, respiratory rate of 18/min, temperature of 96.9°F, and a BMI of 26. A physical exam revealed pupils equal, round, and reactive to light and accommodation; no erythema of the throat; and a supple neck with no lymphadenopathy. The cardiology exam showed a regular rate and rhythm. No edema was present.

Lab work planned at this visit included antinuclear antibody (ANA) screening; C1 inhibitor functional and protein levels; C1Q complement component; total complement; CCP; RF IgG, IgA, and IgM; as well as Smith (SM) antibodies and double-stranded DNA (dsDNA) antibody.

His first reported C1-INH testing was done in 2020 and was negative. His lab results in 2022, after his first rheumatology encounter, also showed negative C1-INH results, with values of 26 mg/dL for protein and 93% for the protein's functional activity. Complement C1Q showed high levels at 9.2. All rheumatoid immunoglobulin factors were elevated, with values of IgG 7 U, IgM 28 U, and IgA 10 U. Normal rheumatoid immunoglobulin factor levels are less than 6 U for IgG, IgM, and IgA. The patient had a high total complement level of greater than 60 U/mL (normal range: 31-60 U/mL), as well as an elevated C-reactive protein of 11.5 mg/L (normal <8 mg/L), as shown in Table 1. All other tests were reported within normal limits.

**Table 1:** Lab results of Patient A with corresponding reference ranges.

Parameter	Patient Values	Reference Range
C1 Inhibitor (Protein)	26 mg/dL	21-39 mg/dL
C1 Inhibitor (Functional Activity)	93%	≥67%
Complement C1q	9.2	5.0-8.6
Rheumatoid Factor - IgG	7 U	<6 U
Rheumatoid Factor - IgM	28 U	<6 U
Rheumatoid Factor - IgA	10 U	<6 U
Total Complement (CH50)	>60 U/mL	31-60 U/mL
C-Reactive Protein (CRP)	11.5 mg/L	<8 mg/L

The patient was prescribed icatibant 30 mg/3 mL, taken subcutaneously with an injector for acute HAE flare-ups, as well as berotralstat 150 mg capsules, taken once daily for the prevention of HAE attacks. The patient's most recent blood work, done in December 2024, showed C1-INH function and protein levels to be within normal limits, as well as complement component C4C. Although the patient continues to experience occasional flare-ups, their frequency and severity have decreased with improved control.

## Discussion

This case highlights the rare presentation of HAE-nC1-INH in a late diagnosis. Despite having symptoms since early adulthood, the patient remained undiagnosed and untreated until age 50. A case study showcasing HAE-induced laryngeal edema demonstrates the risks associated with a late diagnosis. Patient B is a 34-year-old male brought into the emergency department for progressive facial swelling and acute dysphagia [3]. Facial edema developed spontaneously 16 hours prior to presentation, and first-line therapies, such as methylprednisolone and epinephrine, were ineffective, suggesting an alternative diagnosis inconsistent with the initially suspected anaphylaxis. Oropharyngeal computed tomography (OCT) revealed angioedema of the epiglottis with thickening that compromised the patient’s airway [3]. This patient was found to have type I C1 esterase inhibitor deficiency. His family history revealed three generations of idiopathic edema, which is notable for this type of disease. While idiopathic edema is comparable to the case at present, Patient A and Patient B are both manifesting different types of this disease, with Patient A showing no signs of deficient C1-INH on lab results despite recurrent presentations of angioedema. Patient A has not had any obstruction of the airway due to angioedema and does not recall any significant family medical history pertinent to this disease.

A similar case of HAE involved Patient C, a 75-year-old male who had significant breathing difficulty. Patient C presented to the emergency room with an acute onset of facial and throat swelling, accompanied by shortness of breath for the past 10 hours prior to presentation. The patient had an oxygen saturation of 80% and desaturated to 60% during his stay [8]. He admitted to similar recurrent episodes in the past. Patient C did not recall any family history of symptoms similar to his own. The patient was administered an antihistamine and a corticosteroid, both given intravenously, neither of which elicited a therapeutic response [8]. Laboratory results revealed decreased C4 complement level as well as decreased C1 esterase inhibitor function. The patient's symptoms improved once he was placed on a bradykinin inhibitor [8]. Similar to our case with Patient A, both Patients A and C did not have a family history but did have a history of recurrent symptoms, which is pathologic for this disease. Unlike Patient C, Patient A’s C1-INH levels remained within normal limits despite recurrent symptoms. The diagnostic challenges associated with this condition emphasize the need for increased awareness, given its potential for severe clinical consequences, such as the two cases of hypoxia referenced above. HAE-nC1-INH presents a diagnostic challenge due to its reliance on clinical symptomatology and its classification as a diagnosis of exclusion, which, as with many autoimmune diseases, can cause delayed diagnosis. The lack of responsiveness to conventional antihistamine therapy - commonly employed as a first-line treatment for allergic-induced edema - in these patients is associated with an increased risk of adverse clinical outcomes.

**Figure 3:**
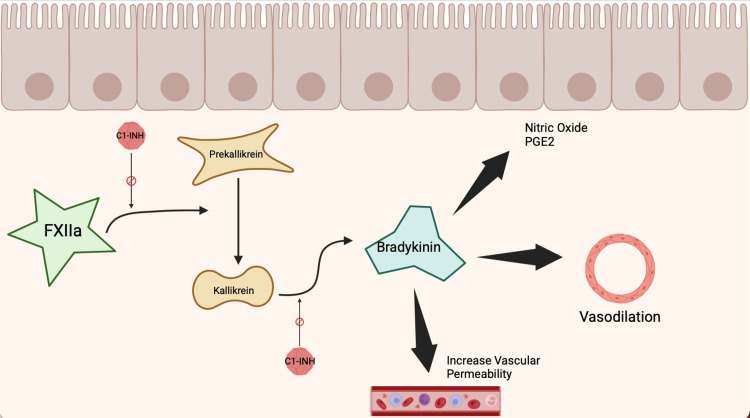
Figure depicts the bradykinin pathway involving activation of factor XII (FXIIa), which converts prekallikrein to kallikrein. C1 esterase inhibitor (C1-INH) regulates the pathway by inhibiting both this conversion and the subsequent formation of bradykinin. Bradykinin promotes vasodilation and increases vascular permeability through the release of nitric oxide and prostaglandin E2 (PGE2). Figure created by © Natalie Elazar, in BioRender [10], using information from [7].

Diagnosis of HAE-nC1-INH relies on clinical features and the exclusion of other causes of angioedema. If the patient has (1) recurrent angioedema without hives, (2) C1-INH levels within normal limits, (3) exclusion of medication-associated angioedema, such as ACEi, nonsteroidal anti-inflammatory drugs (NSAIDs), and tissue plasminogen activator, (4) no response to mast cell therapies, and (5) a response to B2 bradykinin receptor antagonists, then this diagnosis is supported [9]. Some genes thought to be associated with HAE-nC1-INH are F12, FXII, PLG, and ANGPT1. The patient in our case has not had any genetic testing to determine if these genes are present [9]. A limitation of this case report is the absence of genetic testing for known HAE-associated genes, such as F12, PLG, and others mentioned. Variants in these genes have been implicated in HAE-nC1-INH, and their identification can provide important diagnostic confirmation as well as insight into disease subtype. The lack of genetic data limits our ability to fully identify the underlying etiology in this patient. Some treatments for HAE target and antagonize bradykinin release. Drugs such as a plasma-derived, nanofiltered C1-esterase inhibitor and icatibant, a bradykinin receptor antagonist, are examples. Patient A, in our case, was put on berotralstat, a plasma kallikrein inhibitor, as part of the therapeutic regimen, as shown in Figure 3.

## Conclusions

This case study emphasizes the importance of understanding and spreading awareness of the different types of HAE. HAE may be inherited in an autosomal dominant manner; this disease has the potential to affect multiple generations, and, in the absence of timely recognition and accurate diagnosis, it may lead to life-threatening complications. An earlier diagnosis may have been possible with further investigation following the initial negative C1-INH result, along with recognition that repeated, ineffective treatment with corticosteroids and antihistamines warranted alternative therapeutic strategies. This represents a rare case of HAE-nC1-INH that did not result in life-threatening, edema-induced hypoxia; however, numerous cases have demonstrated severe and potentially fatal outcomes. Greater clinical awareness of HAE-nC1-INH will provide physicians and patients with a better understanding of this complex condition, facilitating earlier diagnosis and appropriate management. More investigational research into non-allergic pathways causing angioedema is needed.
